# Machining Path Optimization of Inductively Coupled Plasma Based on Surface Heat Transfer Model

**DOI:** 10.3390/mi16010071

**Published:** 2025-01-08

**Authors:** Peiqi Jiao, Bin Fan, Qiang Xin, Xiang Wu, Hong Wang

**Affiliations:** 1 National Key Laboratory of Optical Field Manipulation Science and Technology, Chinese Academy of Sciences, Chengdu 610209, China; dtjiaopeiqi@163.com (P.J.); wuxiang21@mails.ucas.ac.cn (X.W.); belucky6@163.com (H.W.); 2 Institute of Optics and Electronics, Chinese Academy of Sciences, Chengdu 610209, China; xinqiang@ioe.ac.cn; 3 University of Chinese Academy of Sciences, Beijing 100049, China

**Keywords:** optical processing, heat transfer models, path optimization

## Abstract

Inductively coupled plasma (ICP), a non-contact optical processing method, has been widely used in the preparation of fused quartz. However, the thermal effect during processing inevitably affects the stability of the removal rate, reduces the processing accuracy, and restricts the further development of plasma processing. This paper analyzes the critical temperature that affects the changes in plasma removal depth, establishes a heat transfer model for plasma jet processing through simulations, derives the heat conduction equation during processing, and obtains the critical radius corresponding to the critical temperature related to the processing speed. Additionally, this work analyzes the path temperature of the grating track used in processing and obtains the path temperature variation curve. Based on the critical radius, a staggered grating track was proposed, which verified that this track can effectively control the path temperature, thereby suppressing the error caused by the thermal effect of processing. This study not only helps to gain a deeper understanding of the heat transfer process in plasma machining, but also provides a basis for achieving high-precision plasma machining path optimization schemes.

## 1. Introduction

Fused quartz optical devices, with their excellent optical properties and material uniformity, are not only an indispensable component of typical high-performance optical systems such as Inertial Confinement Fusion (ICF) [1] and Extreme Ultraviolet (EUV) [2], but are also increasingly used in large telescopes, microelectronics, and aerospace [3,4]. With the increasing demand for fused quartz optical devices in optical systems, the technology for efficiently preparing fused quartz has always been a hot topic for scientists. Atmospheric inductively coupled plasma processing is considered a potential solution due to its high efficiency and near-zero damage characteristics [5]. Therefore, many research institutions have conducted research on ICP processing technology [6,7,8].

However, during the ICP processing of the fused quartz surface, a large amount of heat energy will be generated. The change in the surface temperature of the component will lead to nonlinear changes in the material removal rate, which seriously affects the ICP processing accuracy [9,10,11]. In order to reduce the machining errors caused by thermal effects, Castelli et al. developed a process simulation model that considers the spatiotemporal variation in surface temperature and the temperature-related material removal performance [12]. This model can predict the morphology of the machined workpiece. It can guide ICP to achieve a truly deterministic process. Its disadvantage is that the simulation process takes a long time to calculate—about 16–20 h—and it is not convenient to directly iterate and calculate the residence time. In 2012, Mori et al. proposed a reverse staggered raster tool path algorithm considering the thermal compensation coefficient [13]. The algorithm effectively suppressed the influence of thermal effects on the material removal rate during the calculation of the fused silica ICP surface and achieved rapid convergence; however, the selection of the thermal compensation coefficient and the target percentage of material removal used an empirical method. Therefore, it is necessary to establish a more accurate temperature model in the plasma processing process, analyze the temperature change process of the original surface, predict the temperature change in the processing trajectory path through the temperature model, and optimize the processing path more easily and quickly, so as to achieve the purpose of suppressing processing errors.

In this paper, the experiment shows that when the surface temperature of the component is higher than the critical temperature of 340 K, the removal depth of plasma processing will change; therefore, during the processing, ensuring that the temperature of the processing point is lower than the critical temperature of 340 K is the key. Based on the above research objectives, firstly, a heat transfer model of plasma processing was simulated and built, and the effectiveness and accuracy of the model were experimentally verified. Furthermore, the heat transfer equation related to the residence time and the moving speed was derived, and the effective heat transfer radius (critical radius) based on the critical temperature under different parameters was proposed; then, this paper proposed a staggered grating track based on the critical radius, and simulated and compared the traditional grating track. This track can effectively ensure that the temperature of the processing point is lower than 340 K. Finally, the experiment verified that this track, compared to the grating track, can effectively reduce the high-, medium-, and low-frequency processing errors caused by thermal effects. This study provides an important theoretical basis for understanding and explaining the heat transfer of plasma processing, which is conducive to the further development of plasma processing technology.

## 2. Plasma Heat Transfer Research

### 2.1. Heat Transfer Process of Plasma Processing Surface

**Figure 1:**
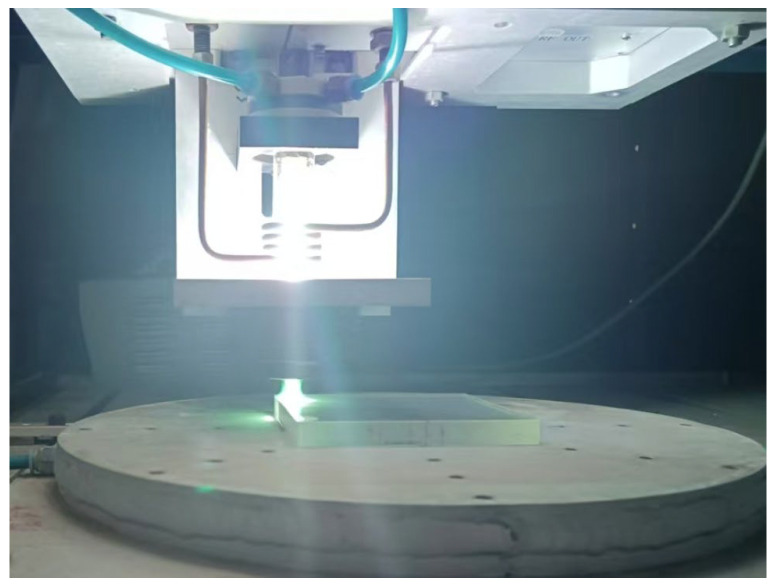
Schematic diagram of plasma processing.

ICP is the process of using a high-frequency power supply to generate a high-frequency current, which is passed into an induction coil. When the high-frequency current passes through the induction coil, an alternating magnetic field is generated around the induction coil. This alternating magnetic field excites the electrons in the gas molecules, ionizing them from atoms or molecules to form plasma. At the same time, the high-frequency electromagnetic field heats the gas, raising the gas temperature, further promoting the ionization process, and forming a stable plasma jet under the action of the electromagnetic field and flow field; material removal is conducted using machining components with plasma jet, as shown in Figure 1.

**Figure 2:**
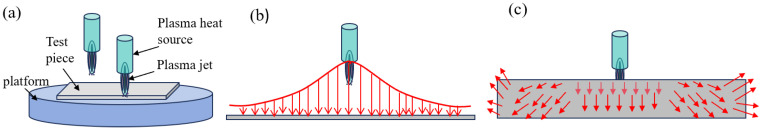
Heat transfer process in plasma processing. (**a**) Processing schematic. (**b**) Schematic diagram of heat transfer from the heat source to the processing starting point. (**c**) Schematic diagram of thermal equilibrium during processing.

The plasma discharge maintenance mechanism is the balance between the absorbed high-frequency electromagnetic field energy and the heat transferred to the outside. On this basis, a stable, high-temperature plasma jet is formed. When the plasma jet contacts the surface of the fused quartz material, the heat is transferred to the surface of the fused quartz material, forming a temperature field. In the plasma fused quartz processing process, heat conduction to balance the process is divided into two stages, as follows:

1. Heat transfer: Heat flows out of the torch nozzle with the plasma and is transferred to the surface of the fused quartz component. Before the plasma jet contacts the surface, the surface is preheated. This process lasts for a short time, as shown in Figure 2b;
2. Thermal balance: When the jet contacts the fused quartz surface, the surface receives heat, forming a heat distribution on the surface, and the heat gradually diffuses into the component. During the diffusion process, the heat is continuously diffused from the fused quartz surface to the air through heat exchange, so that the component cools naturally and finally reaches a thermal equilibrium state, as shown in Figure 2c.

### 2.2. Effect of Temperature on Material Removal Depth

**Figure 3:**
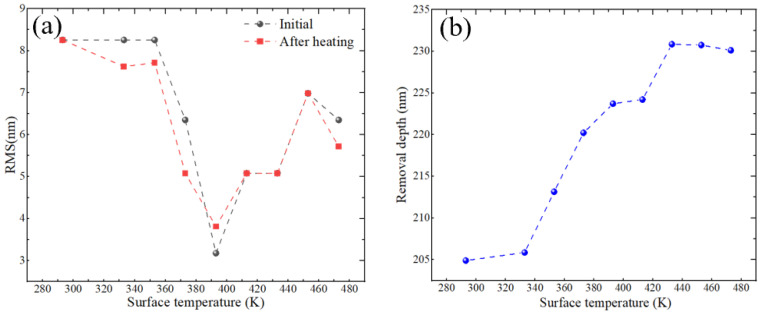
(**a**) Shape changes before and after heating. (**b**) Change in removal depth after heating.

The plasma jet achieves material removal by chemically reacting etching particles with the material surface, and the removal rate is significantly affected by temperature changes [14]. The material removal depth of plasma processing is extracted by detecting the component surface shape before and after processing. Therefore, in order to avoid the influence of heating on the extraction of removal depth, we conducted experiments on a fused quartz element with a diameter of 50 mm and a thickness of 10 mm, and analyzed its surface morphology before and after heating. As shown in Figure 3a, in the absence of plasma etching, the surface shape PV and RMS of the component before and after heating are not affected, eliminating the influence of temperature changes on the surface morphology; Figure 3b shows the change in plasma processing removal depth after preheating the component. It can be seen that when the initial temperature of the component is lower than 340 K, the removal depth remains unchanged; when the temperature is higher than 340 K, the removal depth gradually increases with the increase in temperature. Therefore, during the plasma processing process, we need to keep the average temperature on the component processing path below 340 K to ensure that the removal depth remains unchanged and to reduce the processing error; therefore, 340 K is used as the critical temperature.

### 2.3. Surface Heat Transfer Simulation and Experiment

In order to analyze the heat transfer process of the jet during component processing, the COMSOL 5.6 multi-physics field analysis software was used to construct the heat transfer model. First, the fixed-point heat transfer and mobile heat transfer models were constructed, and the surface component temperature, with a processing power of 400 W, a flow rate of the reaction gas CF4 of 20 mm/min, a processing distance of 12 mm, a dwell time of 5 s, and a line scanning speed of 600 mm/min, was selected for experimental verification. The experiments were carried out on fused quartz with a diameter of 100 mm × 100 mm and a thickness of 10 mm.

**Figure 4:**
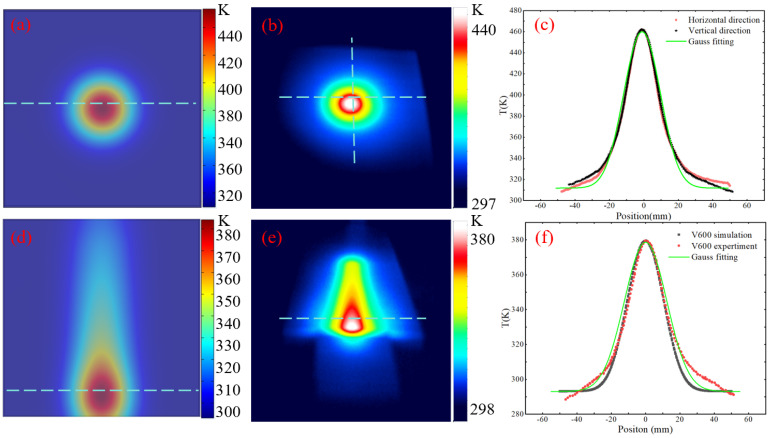
(**a**) Fixed-point heat transfer simulation results. (**b**) Fixed-point heat transfer experimental results. (**c**) Surface temperature extraction and Gaussian fitting of fixed-point heat transfer in horizontal and vertical directions. (**d**) Line scanning heat transfer simulation results. (**e**) Line scanning heat transfer experimental results. (**f**) Horizontal temperature extraction of line scanning core area temperature.

We use the infrared thermal imager of model Testo872 (Testo GmbH, Black Forest, Germany) to detect the surface temperature of the component. Figure 4a is a simulation diagram of fixed-point heat transfer, and Figure 4b is an experimental result diagram. It can be seen that the simulation results are consistent with the experiment. Figure 4c shows the surface temperature extraction along the horizontal and vertical directions during heat transfer. The temperature transfer obeys a Gaussian distribution. Figure 4d shows the simulation results with a moving speed of 600 mm/min; Figure 4e is an experimental result diagram. The results show that as the plasma jet moves along the direction of the scanning path, the core high temperature area is transferred along the moving direction. Figure 4f shows the temperature distribution of the core high-temperature area along the horizontal direction during the movement. The simulation results are consistent with the experimental results and obey a Gaussian distribution.

**Figure 5:**
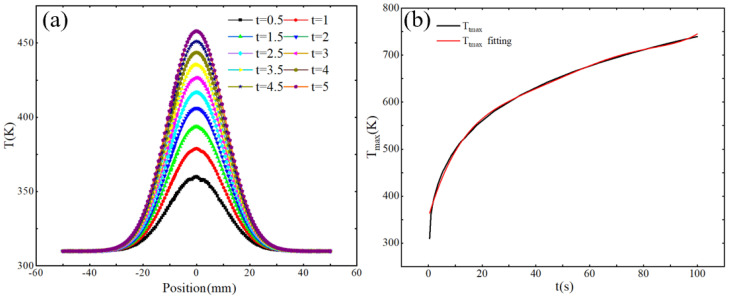
(**a**) Temperature transfer curves at different dwell times. (**b**) Maximum temperature variation with time.

The unification of simulation and experimental results proves the accuracy of the constructed heat transfer simulation model. In order to further analyze the heat transfer law of the plasma processing surface, the heat transfer model of the cumulative heating process is first constructed, and the influence of time changes on the transfer process is analyzed. The results are shown in Figure 5a. As the cumulative heating time increases, the maximum temperature of the surface also gradually increases. The temperature distribution in the entire horizontal direction still conforms to the Gaussian model. The temperature transfer is shown in Equation (1). Figure 5b shows the change and fitting curve of the maximum temperature in the time period of 0–100 s. It can be seen that with the increase in time, the surface will gradually reach a thermal equilibrium state. The polynomial fitting of the maximum temperature change law is shown in Equation (1).

(1) $$T=T_{tmax}{\exp\left(-\frac{2\left(x-x_{c}\right)}{w}\right)}^{2}$$

(2) $$T_{tmax}=354.21+19.39t-0.64t^{2}+0.01t^{3}$$

**Figure 6:**
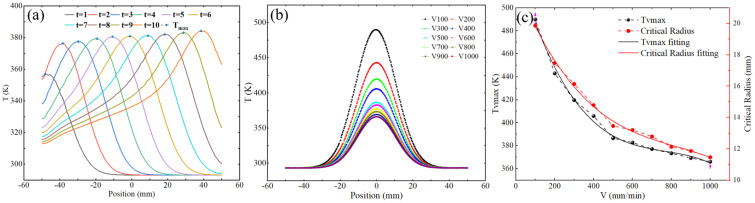
(**a**) Temperature variation curve in the moving direction. (**b**) Temperature variation curve of the core area at different speeds. (**c**) Maximum temperature and critical radius variation curve with speed.

Figure 6a shows the temperature variation trend along the direction of heat source movement when the scanning speed is 600 mm/min. It can be observed that the core high-temperature area moves along the direction of the heat source, and the maximum temperature increases with the movement. This is because the accumulation of heat preheats the next position during the movement of the heat source; in addition, in the moving direction, the temperature of the core area still conforms to the Gaussian heat transfer distribution law. In order to further explore the key factors affecting heat transfer, we simulated the temperature model at different scanning speeds and extracted the transfer curve in the horizontal direction of the same position. As the scanning speed increases, the width of the heat transfer area and the maximum temperature both show a downward trend (as shown in Figure 6b). The results show that the scanning speed has a significant effect on the heat transfer behavior; Figure 6c shows the relationship between the critical radius (CR—defined as the radius of the heat transfer area with a temperature lower than the critical temperature 340 K) and the maximum temperature as the scanning speed changes. The results show that as the scanning speed increases, the CR decreases and the maximum temperature also decreases.

(3) $$T=T_{vmax}{\exp\left(-\frac{2\left(x-x_{0}t\right)}{w}\right)}^{2}$$

(4) $$T_{vmax}=538.37-0.58V+7.21E-4{\times V}^{2}-3.13E-7\times V^{3}$$

(5) $$C_{R}=22.58-0.031V+3.30E-5\times V^{2}-1.33E-8{\times V}^{3}$$

Equation (3) shows the curve equation of heat transfer in the horizontal direction of the core temperature area during the line scanning process as the scanning speed changes. From Equations (4) and (5), the specific values of the maximum temperature and CR of the core area related to the scanning speed can be determined.

### 2.4. Processing Tracks Optimization

**Figure 7:**
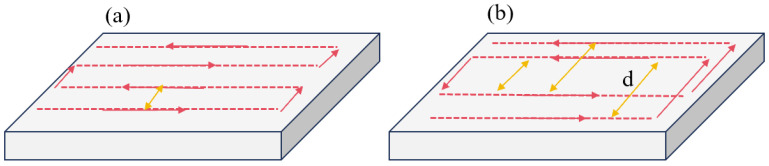
(**a**) Grating track. (**b**) Staggered grating track.

In Section 2.3, a heat transfer model of the component surface during the process of fixed-point heat transfer and the scanning of the plasma jet as a heat source was constructed, and the effectiveness of the model was verified experimentally. As a deterministic optical processing method, plasma processing controls the processing time by adjusting the speed during the process of the surface correction of the optical components to achieve the purpose of different removal amounts at different positions. Its processing trajectory is generally a grating trajectory, as shown in Figure 7a. Therefore, we modeled and analyzed the path temperature of the grating trajectory, using a 100 mm × 100 mm fused quartz component as the base material, a heat source moving speed V of 600 mm/min, a number of paths of 10, and a path spacing of 10 mm.

**Figure 8:**
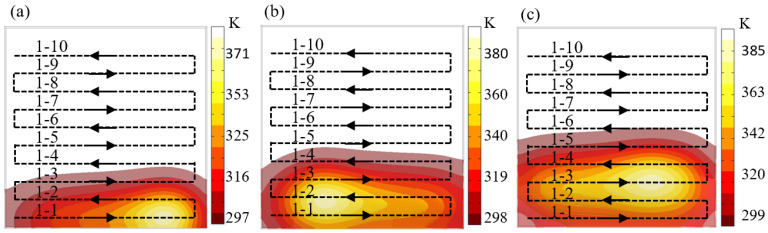
Thermal simulation of grating track. (**a**) The heat source moves on path 1-1. (**b**) The heat source moves on path 1-2. (**c**) The heat source moves on path 1-3.

Figure 8 shows the thermal simulation model of the grating track. It can be seen that the plasma as a heat source moves along the path direction, and the temperature distribution in the core area still obeys the Gaussian distribution. Under the action of surface heat transfer, the initial temperature of the path gradually accumulates and increases, and the maximum temperature of the heat source also gradually increases with the movement process. The accumulation of temperature causes the initial temperature of the rear path to gradually exceed the critical temperature of 340 K. This result causes the material removal depth of the plasma on the rear path to change, thereby causing processing errors. Therefore, how to adjust the path spacing is key to controlling the rise in surface temperature and reducing processing errors.

**Figure 9:**
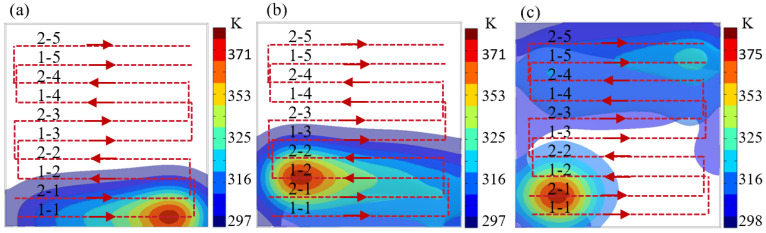
Thermal simulation of staggered grating track. (**a**) The heat source moves on path 1-1. (**b**) The heat source moves on path 1-2. (**c**) The heat source moves on path 2-1.

In the analysis of Section 2.3, the CR at different speeds was obtained. It can be calculated using Equation (5) that when the processing speed is 600 mm/min, the value of the CR is 12.73 mm, and the path spacing of the grating track is smaller than the CR. Therefore, as the heat source moves, the temperature of the path gradually increases, thus exceeding the critical temperature of 340 K. In order to avoid the processing error caused by heat accumulation, a staggered grating track based on the CR is proposed, as shown in Figure 7b. According to the CR corresponding to different processing speeds, the path spacing is adjusted, and in order to ensure the same number of paths and path positions, the track is divided into two parts so that the path spacing of each part is greater than the CR. Heat transfer modeling was performed on the staggered grating track, and the heat transfer of the optimized path was analyzed, as shown in Figure 9.

Figure 9a shows the temperature distribution of the first part of the path (1-1 to 1-2). As the heat source moves along these paths, the temperature gradually increases; however, due to the increase in the path spacing, the superposition effect of the high temperature area is effectively avoided. Figure 9c shows the temperature distribution of the second part of the path (2-1). The distance between path 1-5 and the starting path 2-1 of the second part is much larger than the effective heat transfer distance, so that path 2-1 is effectively cooled and the initial temperature is kept at a low level. In addition, during the movement of the heat source, the path spacing is also kept larger than the CR, avoiding the processing error caused by the excessively high initial temperature. The staggered trajectory design increases the path spacing so that the initial temperature of each path is lower than the critical temperature of 340 K, thereby significantly reducing the processing error caused by the temperature accumulation effect during the movement of the heat source. This optimization effectively controls the heat transfer process and improves the processing accuracy and consistency.

**Figure 10:**
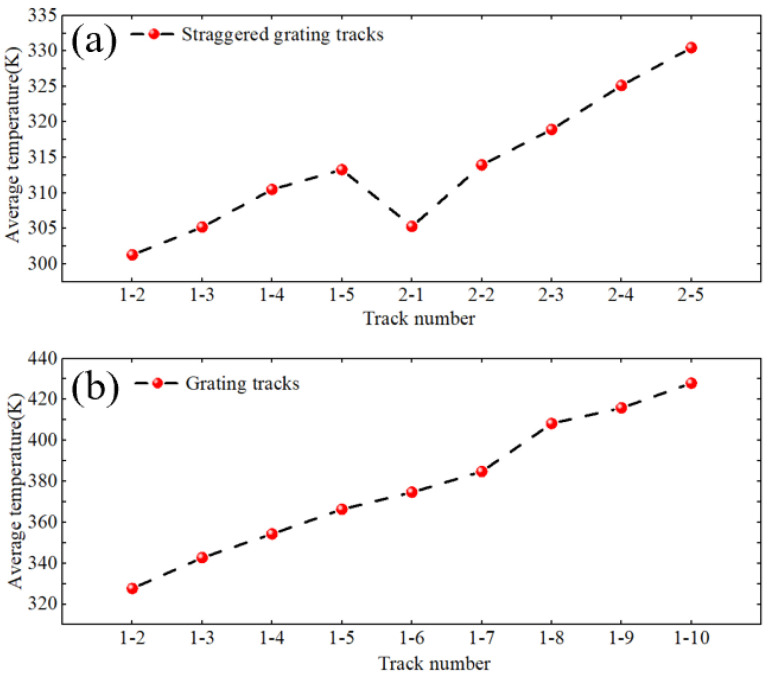
Average temperatures. (**a**) Staggered grating track path. (**b**) Grating track path.

Figure 10 shows the change in the average temperature of the paths under two tracks. As can be seen from Figure 10b, the average temperature of the grating path from path 1 to path 10 shows a gradual upward trend, indicating that as the number of paths increases, the temperature gradually accumulates and increases. Figure 10a shows that the path temperature change in the staggered grating track shows a fluctuating upward trend. Compared with the single grating track, the temperature change in the path of this track is more complex. Each time a new path group starts, the average temperature of the path drops significantly, but as the heat source moves, the temperature still gradually increases, but the average temperature of the path is still below 340 K. The above results show that the staggered grating track can better control the path temperature and avoid the continuous linear temperature increase.

**Figure 11:**
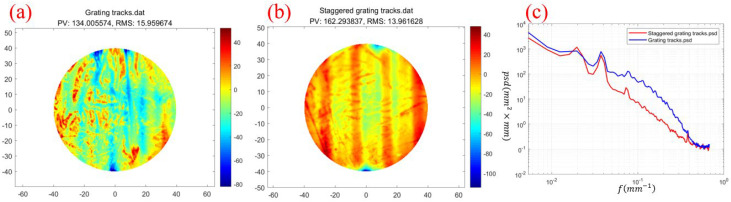
(**a**) The shape after grating track processing. (**b**) The shape after staggered grating track processing. (**c**) The spectrum comparison after the two-track processing.

In order to verify the improvement effect of staggered grating tracks on thermal effect errors, we used two tracks for experimental verification. The experiment used the same initial surface shape. Figure 11a shows the surface morphology after processing with grating tracks. It can be seen that there are many uneven bottom point distributions on the surface, indicating that the removal amount has changed during the movement. Figure 11b shows the surface morphology after processing with staggered grating tracks. Compared with Figure 11a, the area of surface change is more uniform. Figure 11c shows the spectrum comparison of the two tracks. It can be seen that the high- and medium-frequency errors of the staggered grating track are significantly lower than those of the grating track, indicating that the material removal amount can remain unchanged during processing, and the removal amount does not change with the movement of the path. Therefore, the surface shape error after processing is lower than the error caused by the grating track.

## 3. Conclusions

This paper studies the thermal effect of plasma jet processing in the surface processing of fused quartz components by constructing and verifying the jet heat transfer model of plasma processing, proposing an optimized processing path design. The main conclusions and contributions of the study are as follows:

1. The influence of temperature change on the removal depth of the plasma jet components is analyzed, and the critical temperature of the removal amount change is determined to be 340 K.
2. Fixed-point and mobile heat transfer models were constructed, and the accuracy of the heat transfer models was verified experimentally. The results show that the surface temperature distribution of the core heat transfer area of the plasma jet in the fixed-point and mobile processes conforms to the Gaussian model; a temperature simulation model under different heating times and different scanning speeds is constructed, the heat transfer equation related to time and speed is derived, and the corresponding CR under different speeds is analyzed.
3. A temperature model of the grating track in the actual machining process was constructed, indicating that the path spacing is the main factor causing machining errors. The staggered grating track design is proposed. By increasing the path spacing and executing the path in segments, the processing error caused by the temperature accumulation effect during the movement of the heat source is reduced. The experiment verifies that the optimized staggered grating track can effectively reduce the processing error caused by the thermal effect.

## Data Availability

The data presented in this study are available on request from the corresponding author.
